# Active Involvement of End Users When Developing Web-Based Mental Health Interventions

**DOI:** 10.3389/fpsyt.2017.00072

**Published:** 2017-05-03

**Authors:** Derek de Beurs, Inge van Bruinessen, Janneke Noordman, Roland Friele, Sandra van Dulmen

**Affiliations:** ^1^NIVEL, Netherlands Institute for Health Services Research, Utrecht, Netherlands; ^2^Tranzo, Scientific Centre for Care and Welfare, Tilburg, Netherlands; ^3^Department of Primary and Community Care, Radboud University Medical Center, Nijmegen, Netherlands; ^4^Faculty of Health Sciences, University College of Southeast Norway, Drammen, Norway

**Keywords:** user-centered design, online intervention, adolescent health services, design strategies, mental health

## Abstract

**Background:**

Although many web-based mental health interventions are being released, the actual uptake by end users is limited. The marginal level of engagement of end users when developing these interventions is recognized as an important cause for uptake problems. In this paper, we offer our perceptive on how to improve user engagement. By doing so, we aim to stimulate a discourse on user involvement within the field of online mental health interventions.

**Methods:**

We shortly describe three different methods (the expert-driven method, intervention mapping, and scrum) that were currently used to develop web-based health interventions. We will focus to what extent the end user was involved in the developmental phase, and what the additional challenges were. In the final paragraph, lessons learned are summarized, and recommendations provided.

**Results:**

Every method seems to have its trade-off: if end users are highly involved, availability of end users and means become problematic. If end users are less actively involved, the product may be less appropriate for the end user. Other challenges to consider are the funding of the more active role of technological companies, and the time it takes to process the results of shorter development cycles.

**Conclusion:**

Thinking about user-centered design and carefully planning, the involvement of end users should become standard in the field of web-based (mental) health. When deciding on the level of user involvement, one should balance the need for input from users with the availability of resources such as time and funding.

## Introduction

No start-up company today would be around for long if only 5% of its registered customers actually used the product. Also, no start-up would develop a product mainly based on expert knowledge and literature, and then test that product in a 4-year trial. Still, that is the reality of research and practice in the field of web-based mental health interventions (WMHIs) (1, 2). In western countries, mental health disorders such as depression and anxiety are highly prevalent (3). Evidence-based psychological therapies are available on an individual basis, but as this form of treatment is time consuming and expensive, many mental health patients do not receive treatment. WMHIs are thought to improve the accessibility of evidence-based psychological treatment for mental disorders and are becoming increasingly popular (4).

A challenging feature of WMHIs is the high drop-out and non-usage rate (5, 6). A second challenge concerns the implementation and uptake after the initial trial is finished. Often, there is no implementation plan for this last phase, and if the implementation is prepared, the uptake in daily practice seems generally disappointing (2, 7–10). For example, a pragmatic trial examining the implementation of two evidence-based WMHIs [MoodGym (11) and Beating the Blues (12)] found that most participants never used the module or only completed one of the proposed six to eight modules (7). The lack of adherence and poor dissemination does not only hold for WMHIs, but for web-based health interventions in general. Whether in the field of oncology, diabetes (13), or physiotherapy (14), the findings are similar: there is a wide gap between the steady increase in web-based interventions that are being released and their actual uptake. Several factors for the low uptake, such as financial and managerial, have been noted. Another important factor is argued to be the marginal level of engagement of end users during the development phase (10). The more top-down interventions are developed, the more likely they will not match with the needs of the users. As a result, even after research confirmed the effectiveness of web-based health interventions, patients are unlikely to ever use them.

The active involvement of the proposed end user is considered to be an important chance to improve the success rate. With active involvement we mean the collection and usage of input from end users from the start (the design phase) to the end phase (the implementation). Involving end users during the design phase is referred to as user-centered design (UCD). UCD is defined by Preece et al. (15) as “an approach, which views knowledge about users and their involvement in the design process as a central concern.”

The potential benefits of UCD are widely accepted (16). There are a variety of toolboxes available, with methods, tips, and tricks how to involve patients (e.g., *via* http://www.participatiekompas.nl/). The active involvement of patients in the design phase is argued to improve usability and credibility of an eHealth intervention. Usability is defined as the ease a user can use the intervention, and a necessary element to bind end users to an online intervention. Credibility deals with the face-validity and persuasive character of the intervention. It increases the change of acceptance and adherence to an online intervention (17). In practice, however, the actual operationalization, that is, how and when end users are involved, is rarely reported. Therefore, it is also not known how to best involve end users or to what extent adherence is improved when end users are indeed involved.

In this perspective article, we describe the lessons learned from three different web-based health interventions projects that were recently conducted. The projects were selected based on their diversity in development approach and on the experience and knowledge of the authors of this article. The authors do not offer a systematic review or quantitative evaluation but rather share recent insights to stimulate a discourse on user involvement. In paragraph three, lessons learned are summarized, and recommendations provided.

## Different Methods to Develop Web-Based Interventions

In this section, we describe three recent eHealth projects. We start with a short description of the applied method. Next, a health intervention that was developed according to the described method is outlined. Finally, we focus on how the end users were involved.

### Expert-Driven Method

In an expert-driven or top-down method, a small group of specialists usually develops an intervention. They identify a problem or have been asked to develop an intervention for a specific problem by others. After they reach consensus on the problem and the proposed intervention, a first draft of the intervention is developed. This draft is then discussed within the group of experts. If accepted, the intervention is further developed and tested among a few patients or other experts. Based on the feedback, the intervention is adjusted and finalized.

#### Application of Expert-Driven Method: An E-Learning Module for Suicide Prevention

The first author (Derek de Beurs) recently developed and tested an e-learning supported Train-the-Trainer program to implement the suicide prevention guideline in Dutch mental health care (18). In this study, experts in suicide prevention trained senior professionals. Next, these professionals trained their own peer-colleagues in a 1-day face-to-face training session (19). The face-to-face training was supplemented with an e-learning module, as multifaceted interventions have been found to be more effective when compared with single interventions (20, 21). The e-learning module was developed according to an expert-driven method: the research team developed scenarios for six videos to cover the content of the guideline (22). Two experienced nurses and two experienced psychiatrists were approached to act as a role model in the module. After the videos were tapped, one of the researchers edited the videos into 5-min scenes, selecting those parts that reflected the guideline recommendations best. The link to the first version of the e-learning module was sent to the research team and the clinical experts that were role models in the videos. They all commented on the selected videos and specifically on the text that accompanied the scenarios. When all experts agreed to the final version, the module was piloted among a small group of mental health professionals. A short translated demo is available *via* https://www.youtube.com/watch?v=tj9kFrYzynw.

#### Level of User Involvement

The scenarios were developed by the researchers themselves and not presented to professionals before the actual videos were made. One researcher edited the material, deciding himself which scenes were relevant to the end users. The final version was piloted among proposed end users, but only minor adjustments were possible. By not involving end users earlier it was not anticipated that, for example, end users could not view the movies on the workplace because of firewall restrictions, and the professionals interpreted the content of the different scenes ambiguously. As a result, only 23% (122 of the 518 professionals) of the mental health professionals used the e-learning module (18).

### Intervention Mapping (IM)

A method developed to structure the integration of evidence-based information, theory, and practice in the field of health-promoting programs is the IM Framework (23). One of the aims of the IM framework is to develop interventions or programs that are compatible with the targeted population. The framework comprises six fundamental steps and systematically guides the planning and decision-making process. Each step comprises several tasks, and the completion of these tasks guides the subsequent step. It has been used successfully in developing a range of eHealth programs (24–26). The framework starts with a needs assessment or problem analysis (step 1). In step 2, the needs assessment is used to develop the frame of the intervention by defining its objectives. Accordingly, theory-based intervention methods and practical strategies are selected (step 3). By translating the method and strategies, the intervention is than developed in step 4. Step 5 considers the adoption, implementation, and sustainability of the intervention, and in the last step an evaluation plan is developed. Although the process is presented linear and works cumulative, iterative actions are possible.

#### Application of IM: PatientTIME

PatientTIME.nl is a web-based platform aiming to support patients with malignant lymphoma in how to gain more control over the communication with their health-care provider (17, 27). The intervention makes use of different evidence-based methods; video modeling, tailoring information, pre-visit goal setting, and listening back to one’s own audio-recorded visits. Registered patients get a personal secured account with information tailored to their personal needs and characteristics.

The IM framework was used as theoretical backbone of the protocol applied to develop the intervention with corresponding evaluation and implementation plan. IM mapping was chosen as a guideline because it links decisions, final materials, and activities to theory.

In the PatientTIME project, the applied protocol was altered to realize a more patient-driven protocol (17). Practical patient participatory methods were integrated in the theoretical IM framework and used to inspire when and how patients could be involved. A preparatory step was added to the IM framework to plan and prepare the patient participation throughout the entire protocol.

#### Level of User Involvement

The user involvement in PatientTIME (i.e., patients diagnosed with malignant lymphoma) was operationalized in three ways and started early in the project. First, a close collaboration with the patient association for malignant lymphoma was set up. Members of the patient association requested the researchers to develop an intervention for the purpose mentioned above. During the course of the project, the patient organization informed and supported patients and championed patient interests. They were also involved from the start in the development of the implementation plan. Second, two patients were included as research partners who were involved throughout the entire project. They were equal partners next to the researchers and clinicians by having an agenda setting and decision-making role, and their involvement ensured a continuous patient-centered view. Third, 37 patient service users (28 patients and 9 spouses) contributed to this needs assessment. Finally, the usability and credibility of the intervention was thoroughly tested with two patients and two healthy people. They were asked to test the major functionality of the intervention and were encouraged to verbalize their thoughts while testing (17).

In line with other studies, more actively involving patients during the developmental phase resulted in many changes of the intervention that otherwise would not have been made (17). As a concrete example, one of the identified issues was that the presentation of the video clips was unclear. This led the developers and researchers to improve the video diaries. When the intervention was tested in a randomized trial, the patient–program interaction showed that the core element (the video fragments) were well used (28). It seems highly likely that the involvement of patients during the design phase resulted in higher adherence. However, it was not tested if and in what way the more active involvement of patients improved the adherence to eHealth interventions.

### Scrum

A relatively new development method in the field of online health interventions is derived from the agile working principle, originally used in software development (29). Working agile became synonym for an iterative, dynamic, and flexible way of working, involving all stakeholders during the process. One popular agile framework is called scrum, after an element of rugby that involves the team players to pack closely together with their head down in order to get possession over the ball (30). Scrum resembles a difficult task performed by many players working closely together. It is defined as “A framework within which people can address complex adaptive problems, while productively and creatively delivering products of the highest possible value” (30). Scrum shares with science that it is based on empirical data; data are gathered, and decisions are made based on that data.

#### Application of Scrum: Listeningtime

Recently, a web-based intervention for older patients with cancer and their caregivers is developed called “Listeningtime”.^1^ The aim of the intervention is to support patients and their caregivers in reaching effective communication during clinical encounters. The content of Listeningtime is based on the expectations, needs, and experiences of elderly patients^2^ and their caregivers and builds on the earlier discussed PatientTIME intervention. Listeningtime also makes use of short “tailored” video fragments of simulated doctor–patient interactions and an audio-facility to listen back to recorded encounters.

The scrum framework has been used to develop the intervention. Six 2-week sprints with regular meetings between researchers (in this case the product owners), developers, (ex)patients, oncological health-care professionals, and their representatives (i.e., one of a patient and one of a provider organization) were planned. The first sprint started with a “brown paper session,” where the user requirements of end users (i.e., researchers, patients, and oncological health professionals) were mapped. In the following sprints, first features of the platform were built by the developers, and researchers inquired input from potential end users. After a few sprints, it appeared that a sprint session every 2 weeks was not feasibly for the researchers and end users. Sprint frequency was adapted to once every 3 weeks.

#### Level of User Involvement

At the start of the Listeningtime project, potential end users (i.e., patients and health professionals) were interviewed by the researchers (see text footnote 1). Information from this preliminary needs assessment provided input for the first product backlog that was realized during the brown paper session. Parallel to the development of the web-based platform, scripts for the video fragments were written and shared with the end users before any actual video-recordings were made. Between 1 and 14 end users were involved during the sprints. Most end users participated during one sprint, some during several sprint. Their role during the sprints was advising or/and decisional. During the different sprints, the involvement of end users was planned, but their actual attendance was not that easy to realize. To start with, the end users (oncology nurses, oncologists, and older patients diagnosed with cancer) are a difficult group to continually involve due to their high work load or the burden of the disease. Next, the short feedback loops challenged the researchers to interpret the data and adjust the intervention accordingly. More details about Listeningtime and user involvement can be found elsewhere (see text footnote 1).

## Challenges and Considerations When Involving End Users

User involvement during the development process is an important aspect that may influence the successful uptake of a web-based intervention. As we have seen, there are several ways to involve end users. Bellow, we highlight the challenges and considerations when involving end users.

### When to Stop?

Working actively with end users requires that smaller hypothesis are tested in fast feedback loops. As reported in Listeningtime, the sample size for short sprints was mostly small ranging from 1 to 14 people and homogeneous (as mainly male patients with prostate cancer were involved). The major challenge is how to determine if a hypothesis is false or not. UCD is mainly assessed *via* qualitative (interview) methods (31). There are no psychometrically sound assessment tools. When can we say that enough users have given feedback on the product, and the product is ready (enough) to be implemented?

### Involving (Mental Health) Patients and Health-care Professionals

End users for more commercial application, such as a new supermarket app, are quite easy to find, as most people tend to visit supermarkets. When developing a web-based tool for, for instance, depressed patients, it is a challenge to find several groups of 5–10 depressed patients to test prototypes of a web-based intervention. Depending on their mental health condition, it can be quite difficult to actively involve them in the project. When developing products for (mental) health professionals, the time pressure and loss of production limit the availability of end users. Therefore, involvement of mental health patients needs to be carefully planned, which might make short-term development cycles (sprints) less feasible. In PatientTIME, flexibility in terms of planning and setup was experienced as a precondition to get seriously ill patients involved (17).

### Difference in Working Processes

Adjusting a design or changing login procedures is relatively easy to do by experienced developers. Developing, adjusting, and verifying the content of the intervention with researchers and targeted end users are a much slower process. At the start of Listeningtime (2.3), the software developers proposed to meet every week. As this was not feasible for the scientists and the targeted end users, it was agreed to organize the sprints every 2 weeks. Soon it was realized that this interval was also not manageable. Before the first sprint session, time should be invested to understand the different ways of working. In the end, this will save time and frustration.

### Research Proposals As a Starting Point

Traditionally, when applying for a grant to develop a new WMHI, a detailed development, evaluation, and implementation plan is required. This challenges the researcher to let end users influence the development process. Additionally, often only a small portion of the budget is reserved for the actual development phase in the research proposal. We have seen that the involvement of end users takes time. When more feedback loops are incorporated, the budget reserved for the technological development becomes larger. These aspects require a different mind-set from funders, developers, and researchers.

## Conclusion, Limitations, and Future Research

Thinking about user-centered design techniques and carefully planning the involvement of end users should become standard in the field of web-based (mental) health. As this article is based on only three cases and the experiences of the authors, we have not provided an extensive overview of user-centered methods for web-based health interventions. When deciding on the level of user involvement, one should balance the need for input from users with the availability of resources such as time and funding. Further research should provide guidance how to select the best user-centered design strategies for the development of web-based (mental) health intervention.

## Author Contributions

DB and RF initiated the idea. DB and IvB drafted the first version. All authors contributed to the following versions and approved of the final version.

## Conflict of Interest Statement

The authors declare that the research was conducted in the absence of any commercial or financial relationships that could be construed as a potential conflict of interest.

## Footnotes

^1^Noordman J, Driesenaar JA, van Bruinessen IR, van Dulmen S. ListeningTime; participatory development of a web-based preparatory communication tool for elderly cancer patients and their healthcare providers. *Under review* (2017).

^2^Noordman J, Driesenaar JA, Henselmans I, Verboom J, Heijmans M, Dulmen SV. Patient participation during oncological encounters: barriers and need for supportive interventions experienced by elderly cancer patients. (2016).
